# Prediction of CCND1 amplification using plasma DNA as a prognostic marker in oesophageal squamous cell carcinoma

**DOI:** 10.1038/sj.bjc.6605657

**Published:** 2010-04-13

**Authors:** H Takeshita, D Ichikawa, S Komatsu, M Tsujiura, T Kosuga, K Deguchi, H Konishi, R Morimura, A Shiozaki, H Fujiwara, K Okamoto, E Otsuji

**Affiliations:** 1 Division of Digestive Surgery, Department of Surgery, Kyoto Prefectural University of Medicine, 465 Kajii-cho, Kawaramachihirokoji, Kamigyo-ku, Kyoto 602–8566, Japan

**Keywords:** biomarker, plasma, copy number, cyclin D1, oesophageal cancer

## Abstract

**Background:**

We aimed to develop a new biomarker to predict cyclin D1 (CCND1) status using plasma DNA in oesophageal squamous cell carcinoma (ESCC) patients.

**Methods:**

We evaluated the ratio of the CCND1 (11q13) dosage to the dopamine receptor D2 (DRD2; 11q22-23) dosage (C/D ratio) as CCND1 copy number. This study was divided into three steps: (1) Determination of a cutoff value for the C/D ratio in test scale; (2) Comparison of the C/D ratio in between plasma samples and cancer tissues in ESCC patients showing high plasma C/D ratio; (3) Validation study of the clinical application of the plasma C/D ratio as a diagnostic and prognostic marker, by comparing with clinicopathologic factors in 96 ESCC patients.

**Results:**

The plasma C/D ratio was significantly higher in the ESCC group than the controls (*P*=0.0134). A high plasma C/D ratio reflected the tumour C/D ratio, and significantly correlated with a poorer prognosis (*P*=0.0186). Moreover, the high C/D ratio was found to be an independent prognostic factor on multivariate analysis (*P*=0.0266; hazard ratio 5.988).

**Conclusion:**

Prediction of CCND1 amplification using plasma DNA is thought to be a promising prognostic biomarker in ESCC patients.

Oesophageal cancer occurs worldwide with a variable geographic distribution, and ranks eighth in order of occurrence and is the sixth leading cause of cancer mortality (Enzinger and Mayer, 2003). Although there are two different kinds of histologic types of oesophageal cancer, squamous cell carcinoma (ESCC) accounts for ∼90% of all oesophageal cancer diagnosed in Asian countries (Hiyama *et al*, 2007). In spite of the recent advances in diagnostic techniques and perioperative management, patients with ESCC still show extremely poor survival rates even after extended surgery.

Several recent studies have elucidated that certain molecules, such as p53, cyclin D1 (CCND1) and FAS, have important roles in tumourigenesis and the development of the ESCC (Hollstein *et al*, 1990; Adélaide *et al*, 1995; Shinozaki *et al*, 1996; Gratas *et al*, 1998). However, few molecules have been applied in clinical setting as therapeutic and/or diagnostic biomarkers. Therefore, the significance of detecting a novel biomarker using reasonable molecules should be emphasised.

Among several molecules, gene amplification of CCND1 mapped to the 11q13 region, which is a key regulator of the G1 phase of the cell cycle, has been defined as one of the most common genetic aberrations in ESCC, and has been shown to correlate with advanced stage and poor prognosis (Jiang *et al*, 1992; Kyomoto *et al*, 1997). In contrast, recent reports have shown that tumour released circulating DNA and RNA in plasma–serum samples were useful for detecting the tumour-specific abnormalities and monitoring disease status in cancers (Sozzi *et al*, 2003; Kimura *et al*, 2006; Skvortsova *et al*, 2006; Diehl *et al*, 2008; Schwarzenbach *et al*, 2009).

Therefore, we hypothesised that the genome status of CCND1 in primary oesophageal cancer could be predictable by a less invasive, blood-based test, which enable to evaluate primary tumour dynamics and predict prognosis and associated clinicopathological factors. To circumvent the influences of plasma DNA quantity and numerical chromosomal aberrations, we particularly calculated the ratio of the dosage of CCND1 to that of the reference gene, dopamine receptor D2 (DRD2) gene mapped to 11q22-23, as an indicator of the CCND1 copy number (CCND1/DRD2: C/D ratio) (Kyomoto *et al*, 1997).

In this study, we showed whether the plasma C/D ratio is useful to predict tumour CCND1 amplification in preliminary 40 ESCC patients, and examined the diagnostic value of the C/D ratio to detect cancers by comparing findings in ESCC patients and healthy volunteer controls. In addition, we validate the prognostic value of the C/D ratio by analysing the relationship between the plasma results and clinicopathological factors in 96 consecutive ESCC patients.

## Materials and Methods

### Patients and samples

Between October 2007 and April 2009, recent 40 preoperative plasma samples were collected from consecutive patients with ESCC, who underwent R0 (no residual tumour after surgery) or R1 (microscopic residual tumour after surgery) esophagectomy at the Kyoto Prefectural University of Medicine, as well as from 19 healthy volunteers. After determination of the cutoff value for the plasma C/D ratio (see the section ‘Quantitative analysis for CCND1 using real-time polymerase chain reaction’ described below), we divided the 40 patients into two groups, 19 patients with a high plasma C/D ratio and 21 with a low plasma C/D ratio. Paraffin-embedded oesophageal tumour tissues and adjacent normal oesophageal tissues were collected from the same 19 patients showing the high plasma C/D ratio. To obtain additional evidence that the tumour-derived circulating DNA reflect the plasma C/D ratio, the postoperative plasma samples (20–51 days after operation) were also collected from eight patients in the high plasma C/D ratio group, who underwent R0 esophagectomy.

Plasma samples were collected from another 56 consecutive ESCC patients and another 21 healthy volunteers for large-scale validation assays. In total, plasma sample were collected from 40 healthy volunteers and 96 consecutive ESCC patients who underwent curative surgery (R0 or R1) between April 2005 and April 2009.

Resected cancer specimens were fixed in buffered formalin and embedded in paraffin for routine pathological examination. Pathological classification of the tumour was determined according to the UICC classification (Sobin and Fleming, 2002).

### Preparation and DNA isolation of plasma and tissues samples

After obtaining informed consent, 7 ml of peripheral blood was obtained from each patient before any surgery, and from healthy volunteer controls. Immediately after collection, the blood samples were subjected to the isolation of cell-free nucleic acids by the three spin protocol (1500 r.p.m. for 30 min, 3000 r.p.m. for 5 min, 4500 r.p.m. for 5 min) to prevent cross contamination from cellular nucleic acid (Tomita *et al*, 2007). After centrifugation, the plasma sample was stored at −80 °C until further processing. Cell-free genomic DNA was isolated from 400 *μ*l of the serum sample using the QIAamp blood mini kit (Qiagen, Hilden, Germany). The final elution was performed using a 100 *μ*l of the AE buffer of the QIAamp blood mini kit.

Paraffin-embedded tissues were sectioned at 5 *μ*m thickness after undergoing routine histologic examination. Genomic DNA of cancerous and adjacent noncancerous oesophageal tissues were extracted from the two slices 5 *μ*m thick using DEXPAT (TaKaRa, Kyoto, Japan) according to the manufacturer's protocols.

### Quantitative analysis for CCND1 using real-time PCR

We selected the DRD2 gene as a reference gene, because the DRD2 is located on the same chromosome as CCND1, and DRD2 amplification and overexpression in ESCC have never reported in any gene database, such as GenBank, DDBJ and EMBL. Therefore, the CCND1/DRD2 ratio would reflect the CCND1 copy number without influence of absolute DNA concentration and aneuploidy of chromosome 11.

The serum dosage of CCND1 and DRD2 were quantified by DNA-based real-time PCR using Applied Biosystems 7300 Real-Time PCR system (Applied Biosystems, Foster City, CA, USA). The PCR mixture was a final volume of 20 *μ*l containing 10 *μ*l TaqMan Universal PCR Master Mix (Applied Biosystems), 1 *μ*l TaqMan Gene Expression Assays and 9 *μ*l each DNA samples. TaqMan Gene Expression Assays for CCND1 (Hs03772544_cn) and DRD2 (Hs05266854_cn) were purchased from Applied Biosystems. All PCR reactions were performed with one cycle of 95 °C for 10 min, followed by PCR amplification with 40 cycles of 95 °C for 15 s and 60 °C for 60 s.

The CCND1 copy number of DNA samples was determined by the ratio of the CCND1 dosage to the DRD2 dosage (C/D ratio), using the comparative threshold cycle method.

### Statistical analysis

The difference in the plasma C/D ratio between the ESCC and healthy volunteer control groups was assessed using the Mann–Whitney *U*-test, and that between preoperative and postoperative findings was assessed using Wilcoxon *t*-test. *χ*^2^ test, Fisher's exact probability test was used to evaluate the correlations between results of the plasma C/D ratio and clinicopathological factors. *P-*values <0.05 were considered statistically significant. Moreover, overall survival rate was calculated using Kaplan–Meier method, and the significance of differences was determined by log-rank test. Multivariate stepwise Cox regression model was performed to identify significant contributors that were associated independently with death on univariate analysis; multivariate risk ratios are presented with 95% confidence intervals.

## Results

### Study design to develop a novel genomic biomarker of the plasma

**Figure 1:**
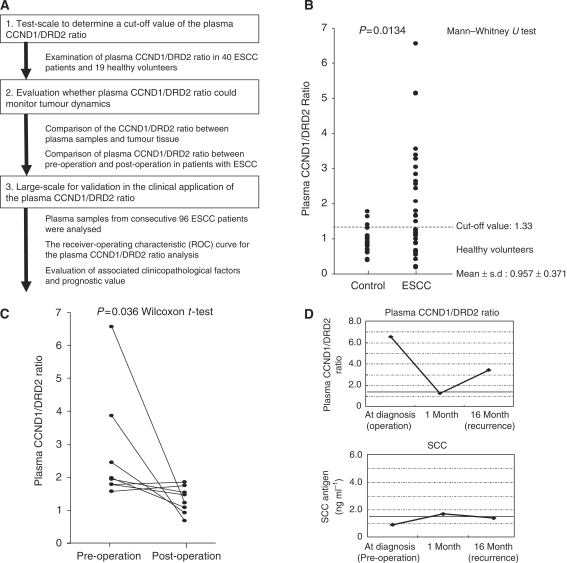
Study design and initial results to develop a novel genomic biomarker of the plasma. (**A**) Strategy to identify novel plasma potential biomarker in ESCC. (**B**) Comparison of plasma CCND1/DRD2 (C/D) ratio in 40 ESCC patients and 19 healthy volunteers by real-time PCR. The plasma CCND1/DRD2 ratio was significantly higher in the ESCC group (median: 1.24, range: 0.19–6.56) than in the control group (median: 1.05, range: 0.39–1.79). (**C**) Comparison of plasma CCND1/DRD2 ratio between pre- and postoperative plasma. The plasma CCND1/DRD2 ratio was also examined postoperatively in eight patients showing a high plasma CCND1/DRD2 ratio in preoperative plasma, and the value was significantly reduced in postoperative plasma (*P*=0.0357, Wilcoxon *t*-test). (**D**) Changes of the plasma CCND1/DRD2 ratio and SCC antigen in one patient who developed recurrences. The top and bottom graphs represent the value of the plasma CCND1/DRD2 ratio and SCC antigen level, respectively. The dotted lines represent the upper limit of each marker: 1.33 for plasma CCND1/DRD2 ratio and 1.5 ng ml^–1^ for SCC antigen. The patients underwent esophagectomy, however, radiologic examination showed multiple recurrences, lymph nodes and lungs, 12 months postoperatively. The plasma CCND1/DRD2 ratio re-elevated at the time of diagnosis of recurrences.

The study design is summarised in Figure 1A. This study was divided into three steps: (1) Determination of a cutoff value of the C/D ratio by comparing results of both plasma from 40 ESCC patients and 19 healthy volunteers; (2) Evaluation whether plasma C/D ratio could monitor tumour dynamics; (3) Validation study for clinical application as a diagnostic and prognostic marker, using the plasma C/D ratio, by comparing the plasma results with clinicopathologic factors in 96 ESCC patients.

### Test scale for detection cutoff value of plasma CCND1/DRD2 ratio

Plasma C/D ratio of 40 ESCC patients and 19 healthy volunteer controls was examined by real-time PCR to determine the cutoff value. Figure 1B shows the distribution of the plasma C/D ratio in ESCC patients and in the control groups. The plasma C/D ratio was significantly higher in the ESCC patients group than control group (median: 1.23 (range 0.19–7.32) *vs* median: 1.05 (range 0.39–1.79), *P*=0.0134). Consequently, we determined a cutoff value of 1.33 for the plasma C/D ratio based on the mean value + s.d. in healthy volunteer controls to distinguish the high C/D ratio patients from those with low ratio patients. As a result, 19 patients were categorised into high plasma C/D ratio group, whereas 21 patients in low plasma C/D ratio group.

### Evaluation whether plasma CCND1/DRD2 ratio could monitor tumour dynamics

We analysed the C/D ratio of oesophageal cancer and adjacent normal oesophageal tissues in 19 ESCC patients showing a high plasma C/D ratio. The higher C/D ratio of oesophageal tumour compared with that of normal tissues was found in 14 of the 19 patients (74%) (Table 1), while a higher C/D ratio of oesophageal tumour was found in only 6 of the 21 ESCC patients showing a low plasma C/D ratio (Supplementary Table 1). These findings indicated that the high plasma C/D ratio reflects the amplification in tumour. Moreover, we examined the CCND1 assay in postoperative plasma of eight patients showing high plasma C/D ratio in preoperative plasma. The C/D ratio reduced significantly in the postoperative plasma samples (*P*=0.0357; Figure 1C). In one patient, the re-elevation of plasma C/D ratio was found at 16 month after surgery, who developed recurrence without elevation of conventional serum tumour marker such as squamous cell carcinoma antigen (SCC) (Figure 1D).

### Large scale for validation in the clinical application as a diagnostic and prognostic biomarker

**Figure 2:**
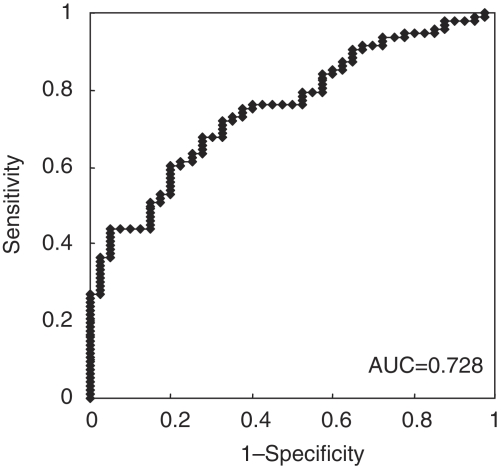
The receiver-operating characteristic (ROC) curves for the plasma CCND1/DRD2 ratio analysis in 96 ESCC patients and 40 healthy volunteers. The area under the curve (AUC) was 0.728 in 96 ESCC patients *vs* 40 healthy volunteers.

In total, 96 consecutive patients were included in this study; the mean age of patients was 64.2 years (range: 44–82), the male–female ratio was 3.4 : 1, and 9 with TNM stage 0, 23 with stage I, 35 with stage II, 21 with stage III and 8 with stage IV. The mean follow-up period was 13.8 months (range: 0–40). Figure 2 shows the receiver-operating characteristic curves for plasma C/D ratio analysis, and the area under the curve was 0.728. Sensitivity was 43.7% (42 out of 96) and specificity was 87.5% (35 out of 40) (Supplementary Figure 1).

Correlations between plasma C/D ratio and clinicopathological factors in 96 ESCC patients are summarised in Table 2. The patients showing high plasma C/D ratio tended to have lymph node metastasis although the difference was not significant. There was no significant correlation between the plasma C/D ratio and other clinicopathological factors in this study.

**Figure 3:**
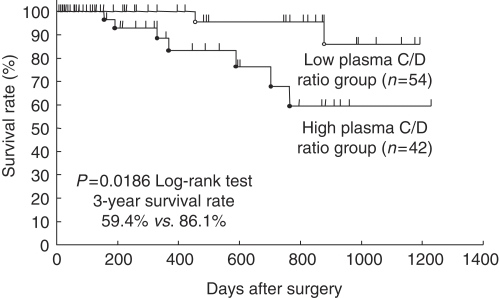
Comparison survival curves according to plasma CCND1/DRD2 (C/D) ratio in 96 consecutive patients with ESCC. Patients with a high plasma C/D ratio (1.33⩽) showed significantly poorer survival than those with low ratio (*P*=0.0186).

We additionally validated the plasma C/D ratio as a prognostic value in 96 ESCC patients. Patients in the high plasma C/D ratio group showed significantly poorer survival than those in the low ratio group (3-year survival rates: 59.4 *vs* 86.1%, *P*=0.0186) (Figure 3). Univariate analysis showed that the T-stage was also a significant prognostic factor. Both the plasma C/D ratio (*P*=0.0266 hazard ratio 5.988 (range: 1.232–29.411)) and T-stage were found to be independent prognostic factors on multivariate analysis (Table 3).

## Discussion

A noninvasive assay using circulating nucleic acids opens up a new and interesting field in the screening and monitoring of cancer patients. Several investigators have recently reported that the detections of tumour-derived circulating DNA and RNA would be promising diagnostic and prognostic tools in clinical practise (Johnson and Lo, 2002; Fleischhacker and Schmidt, 2007). A majority of these approaches, however, require complicated processing including certain modifications. Furthermore, strict handling is required in cases of mRNA assays.

However, gene amplification is one of the most frequent genomic aberrations involved in the pathogenesis of various cancers. Especially in ESCC, oncogenes, such as SMYD2 (1q32.3-q41), EGFR (7p12), MYC (8q24.21), CCND1 (11q13), cIAP1 (11q22) and ERBB2 (17q21.1), have already been identified as major amplification targets associated with development, progression and metastasis of aggressive disease (Lu *et al*, 1988; Imoto *et al*, 2001; Jain *et al*, 2007; Komatsu *et al*, 2009; Sato-Kuwabara *et al*, 2009). Among them, CCND1 is the gene most frequently amplified (22–65%) with high copy numbers, and also associated with a poorer survival outcome in ESCC patients (Lam, 2000). These findings prompted us to detect the CCND1 amplification in plasma samples of ESCC patients, as well as to examine the clinical usefulness of the noninvasive assay as a possible biomarker.

To circumvent the aneuploidy of chromosomes, we first developed real-time quantitative PCR with a reference gene mapped to the same chromosome, and subsequently showed that the ratio, CCND1 (11q13) / DRD2 (11q22-23), in plasma DNA is a valuable diagnostic tool for detecting cancers, monitoring therapeutic effects and predicting both clinicopathological factors and postoperative prognosis of ESCC patients. We analysed whether the plasma C/D ratio could monitor tumour dynamics by two different analyses in advance. One is the comparison between the plasma C/D ratio and amplification in tumour, which showed that the tumour C/D ratio was high compared with that of adjacent normal tissue in most patients with a high plasma C/D ratio. The reasons for the discrepancies in some patients remain to be identified; however, one possible explanation for this finding is the heterogeneity of the primary tumours. Another analysis involves comparison of the plasma C/D ratio in paired plasma obtained before and after surgery. As a result, the plasma C/D ratio was significantly reduced postoperatively in patients who underwent R0 esophagectomy, suggesting that tumour-derived circulating DNA reflect the plasma C/D ratio.

The plasma copy number assay presented in this study has several possibilities for clinical application. First is the screening method in the high-risk group of patients. This assay could detect an elevated C/D ratio regardless of disease stage (detection rate; stage 0–II *vs* III–IV: 42.8 *vs* 48.2%) and tumour depth (T0–T2 *vs* T3–4: 42.1 *vs* 46.2%). These findings suggest that the tumour could release a significant amount of genomic DNA into systemic circulation even at an early stage, and also copy number gains (10–100 copy) derived from CCND1 amplification of each cancer cell chromosome is drastic. This phenomenon was reported in other studies as well as our previous studies showing that the concentration of plasma DNA in cancer patients was significantly higher than that in controls, regardless of tumour stage (Sai *et al*, 2007; Tomita *et al*, 2007). Therefore, circulating DNA in peripheral blood may be an early event in the carcinogenesis of solid cancers, and allow the monitoring tumour dynamics as well as prediction of both associated clinicopathological factors and the prognosis at an early stage.

The second possibility is the clinical application as a marker to monitor therapeutic efficacy (Figure 1C) and recurrence as a complement to conventional serum tumour markers, such as SCC and CEA. In this study, the plasma C/D ratio decreased to the normal range after surgery and increased again at recurrence without any change in conventional serum tumour markers (Figure 1D). In addition, the most interesting finding to be emphasised was that this assay could also be useful for predicting patients with poor prognoses. This is the first plasma–serum copy number study to predict the prognosis in cancer patients, although the copy number of the amplified genes in plasma or serum has been evaluated in some previous studies (Chiang *et al*, 1999; Gotoh *et al*, 2005; Park *et al*, 2009).

Other benefits of this assay include the savings of time and effort compared with those required for other plasma–serum assays. TaqMan real-time PCR assay allows us to easily evaluate the copy numbers of plasma amplified genes in a couple of hours. Previously, we reported that methylation-specific PCR (MSP) assays using circulating DNA could be combined to serve as a tumour marker in gastric cancer (Ichikawa *et al*, 2004; Koike *et al*, 2004). We also showed that the quantification of circulating mRNA such as hTERT and MUC1 using RT–PCR would be useful for the early detection of primary and recurrent gastric cancers (Tani *et al*, 2007). However, these assays are time-consuming, and required strict conditions and handlings. Besides, the detection rates of MSP and quantitative mRNA assays are relatively low (15–30%). Our present plasma copy number assay is more suitable for clinical application from the perspective technical simplicity, rapidity and reliability.

We present here a framework to assess tumour characteristics by noninvasive plasma assay. Recent advances in molecular technologies have allowed the analysis of numerous gene and chromosomal aberrations within the entire genome. In the near future, we could design a ‘tailor-made biomarker’ assay, based on exhaustive evaluation of genes amplifications in primary tumours. This strategy is currently under evaluation.

In conclusion, this study clearly showed that plasma-based CCND1 copy number provides a valuable biomarker for monitoring treatment efficacy and predicting both the associated clinical factors and prognosis of ESCC. This technology could facilitate clinical decision making and be applicable to tailor-made medicine for each individual.

## Figures and Tables

**Table 1:** Evaluation of tumour CCND1 amplification in high plasma CCND1/DRD2 ratio group

		**Tumour CCND1**	
	**Number**	**Amplification**	**Non-amplification**
High plasma CCND1/ DRD2 ratio group	19	14 (74%)	5 (26%)

Abbreviations: CCND1=cyclin D1; DRD2=dopamine receptor D2.

**Table 2:** Correlation between clinicopathological factors and the plasma CCND1/DRD2 ratio in consecutive 96 patients with ESCC

		**Plasma CCND1 amplification value**		
**Variable**	***n***	**Low**	**High**	***P-*value^b^**
Total	96	54 (56.3%)	42 (43.7%)	
				
*Sex*				
Male	74	44 (59.5%)	30 (40.5%)	0.2450
Female	22	10 (45.5%)	12 (54.5%)	
				
*Age*				
<65	42	24 (57.1%)	18 (42.9%)	0.8764
65≦	54	30 (55.6%)	24 (44.4%)	
				
*Lymphatic invasion*				
Negative	67	40 (59.7%)	27 (40.3%)	0.3001
Positive	29	14 (48.3%)	15 (51.7%)	
Venous invasion				
Negative	58	30 (51.7%)	28 (48.3%)	0.2694
Positive	38	24 (63.2%)	14 (36.8%)	
				
*T stage* a				
T0/1/2	57	33 (57.9%)	24 (42.1%)	0.6945
T3/4	39	21 (53.8%)	18 (46.2%)	
				
*N stage* a				
N0	54	35 (64.8%)	19 (35.2%)	0.0550
N1	42	19 (45.2%)	23 (54.8%)	
				
*Stage* a				
0–II	67	39 (58.2%)	28 (41.8%)	0.5564
III–IV	29	15 (51.7%)	14 (48.3%)	
				
*Recurrence*				
Negative	72	44 (61.1%)	28 (38.9%)	0.0963
Positive	24	10 (41.7%)	14 (58.3%)	

Abbreviations: CCND1=cyclin D1; ESCC=oesophageal squamous cell carcinoma; DRD2=dopamine receptor D2.

a TNM classification.

b *P-*values are from *χ*^2^ or Fisher’s exact probability test and were statistically significant at 0.05.

**Table 3:** Cox proportional hazard regression analysis for overall survival in 96 patients with ESCC

	**Univariate** a	**Multivariate** b		
**Factor**	***P-*value**	**HR**	**95%CI**	***P-*value**
*Sex*				
Male *vs* female	0.8596		—	
				
*Age*				
<65 *vs* 65≦	0.6148		—	
				
*Lymphatic invasion*				
Negative *vs* positive	0.7233		—	
				
*Venous invasion*				
Negative *vs* positive	0.1472		—	
				
*T stage*				
T0/1/2 *vs* T3/4	**0.0430**	4.274	1.055–17.241	**0.0419**
				
*N stage*				
N0 *vs* N1	0.6650		—	
				
*Plasma CCND1/DRD2 ratio*				
<1.33 *vs* 1.33≦	**0.0186**	5.988	1.232–29.411	**0.0266**

Abbreviations: CCND1=cyclin D1; CI=confidence interval; DRD2=dopamine receptor D2; ESCC=oesophageal squamous cell carcinoma; HR=hazard ratio.

a Kaplan–Meier method, and the significance of difference was determined by log-rank test.

b Multivariate survival analysis was performed using Cox’s proportional hazard model.

Statistically significant values are in bold face type.

## Supplementary Material

Supplementary Figure legend

Supplementary Table1
