# Supplementary figures and images for: Prediction of CCND1 amplification using plasma DNA as a prognostic marker in oesophageal squamous cell carcinoma
Source: Br J Cancer. 2010 Apr 13;102(9):1378–83. doi: 10.1038/sj.bjc.6605657 (PMC2865765; doi:10.1038/sj.bjc.6605657)

## Slide 1
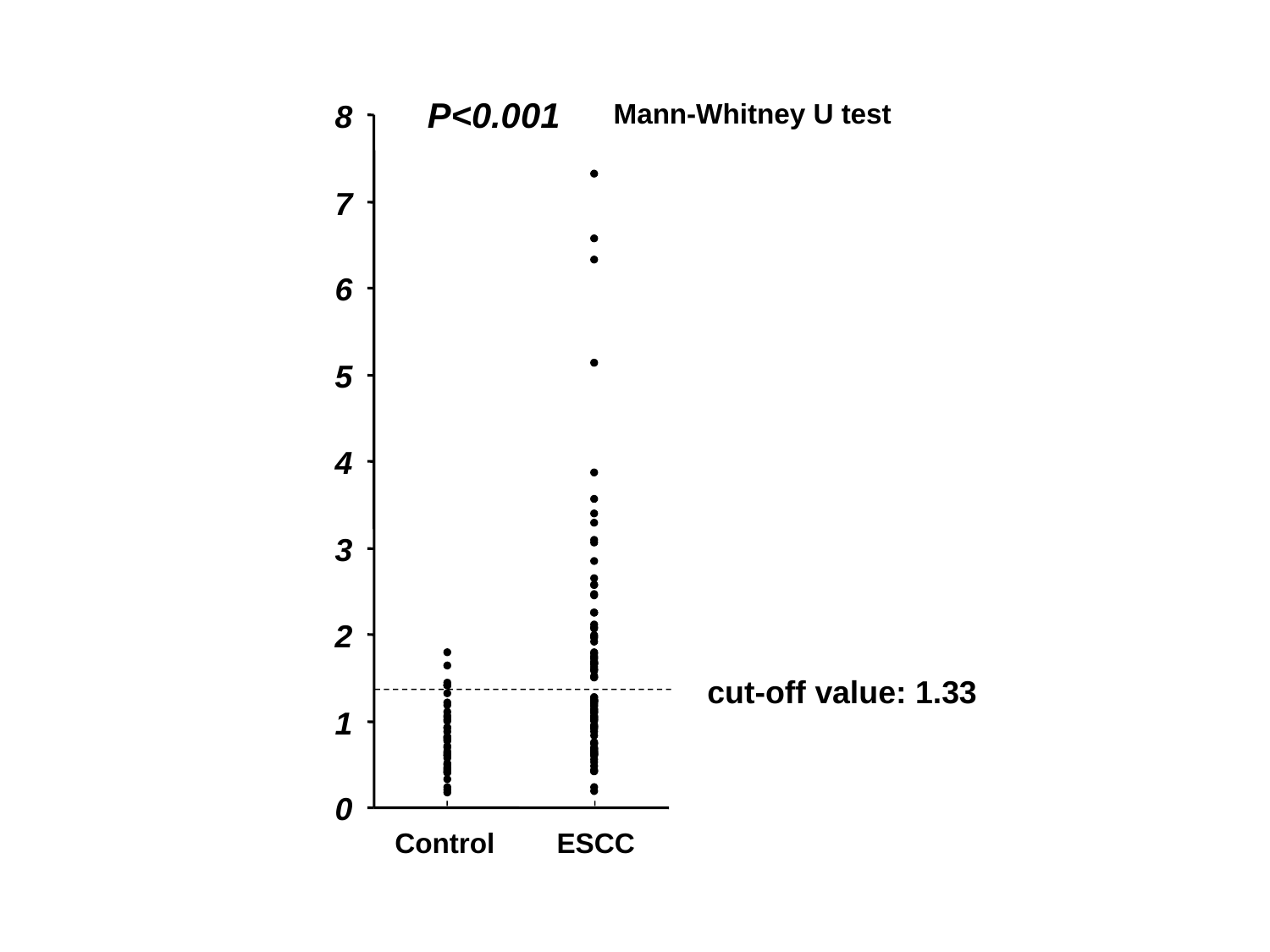

P<0.001
Mann-Whitney U test
8
7
6
5
4
3
2
1
0
cut-off value: 1.33
Control
ESCC

Supplement: Supplementary Figure [file 6605657x1.ppt]
