# Supplementary material for: Prediction of CCND1 amplification using plasma DNA as a prognostic marker in oesophageal squamous cell carcinoma
Source: Br J Cancer. 2010 Apr 13;102(9):1378–83. doi: 10.1038/sj.bjc.6605657 (PMC2865765; doi:10.1038/sj.bjc.6605657)
Supplement: Supplementary Figure legend [file 6605657x2.doc]

**Supplementary Figure 1**

**Comparison of plasma CCND1/DRD2 (C/D) ratio in 96 ESCC patients and 40 healthy volunteers by real-time PCR**, The plasma CCND1/DRD2 ratio was significantly higher in the ESCC group (median: 1.23, range: 0.19 to 7.32) than in the control group (median: 0.76, range: 0.17 to1.79). Sensitivity was 43.7% (42/96) and specificity was 87.5% (35/40).
